# Suitability of lectin binding studies for the characterization of redox-active microbial environmental biofilms

**DOI:** 10.1186/s13568-022-01479-7

**Published:** 2022-11-05

**Authors:** Pablo Ingino, Kai Hao Tiew, Martin Obst

**Affiliations:** 1 BayCEER, University of Bayreuth, Dr-Hans-Frisch-Str. 1-3, 95448 Bayreuth, Germany; 2 School of Science, Monash University Malaysia, Subang Jaya, Malaysia

**Keywords:** Biofilm, Redox potential, Glycoconjugates, Lectin, EPS, FLBA

## Abstract

Aquatic environmental microbial biofilms grow in a broad range of redox environments from oxic to methanogenic, and they often also establish internal redox gradients. In technical applications, biofilms are also subjected to controlled redox conditions. Studies on biofilms often make use of fluorescence microscopic imaging techniques together with lectin binding analysis to gain insights into structure, composition, and functions of the biofilms. Here we studied the direct influence of redox potentials on fluorescence lectin binding analyses (FLBA) for two commonly used lectin-fluorophore conjugates. An effect of the electrical potential on signal intensity was observed and found to be statistically significant. The signal intensity changes however, remained within the range of a few percent total. A significant drop in intensity was only observed for extremely oxidizing potentials, typically not found under environmental conditions. Our results showed that the fluorophore itself and not the lectin binding to the respective glycoconjugate causes fluorescence changes. The two tested lectin-fluorophores are shown to be suitable for studying the distribution and composition of EPS in environmental biofilms or technical applications and under varying redox conditions.

**Supplementary Information:**

The online version contains supplementary material available at 10.1186/s13568-022-01479-7.

## Introduction

In situ imaging techniques such as confocal laser scanning microscopy (CLSM) allow analysis of biofilms in their natural state (Neu et al. 2010). One common approach is fluorescence lectin-binding analysis (FLBA). It uses lectin-conjugated fluorophores to identify and to map the composition of microbial extracellular polymeric substances (EPS). Introduced to a biofilm sample, lectins can bind to their target mono- or oligosaccharide units present in polysaccharides and other glycosylated components, which makes them ideal to label the EPS of biofilms. Hence, FLBA allows for qualitative and quantitative analysis of the EPS matrix (Neu and Lawrence 2016). In the past FLBA helped identify micro domains within biofilms (Lawrence et al. 2007), as well as elucidating the role of environmental parameters on biofilm development (Proia et al. 2012), and interactions with environmental contaminants (Lawrence et al. 2001) and heavy metals (Lawrence et al. 2004; Yang et al. 2011).

Microbial activities in biofilms lead to physical and chemical gradients in space and time (pH, redox potential, and oxygen) (de Beer et al. 1994; Hunter and Beveridge, 2005; Snider et al. 2012). Such gradients allow for a vast diversity of cell types to coexist and result in a high degree of heterogeneity on the spatial and temporal scale (Stewart and Franklin 2008). The formation of microenvironments can lead to bio- and geochemical reactions that seem thermodynamically infeasible when looking at the bulk scale (Gieseke et al. 2006; Li and Bishop 2004). Combining FLBA with additional sensors, such as for metals (Hao et al. 2016, 2013) or pH (Hegler et al. 2010; Hunter and Beveridge 2005) further increases our mechanistic understanding of such microenvironments. However, correlating the signals gained from FLBA to other sensors requires an environmental insensitivity of the lectin-fluorophore conjugates used. Fluorescein and its derivatives for example are known for their pH-sensitive fluorescence (Lanz et al. 1997; Martin and Lindqvist 1975; Sjöback et al. 1995). Thus, lectins are often conjugated with fluorophores of the Alexa family to stain EPS, as they exhibit a wide pH stability (Panchuk-Voloshina et al. 1999). However, potential dependent redox switching of organic fluorophores was observed as well (Lei et al. 2009; Salverda et al. 2010).

Environmental biofilms exhibit a wide range of redox potentials, ranging from methanogenic, sulfidic, iron, manganese, and nitrate reducing to completely oxic conditions (Kappler et al. 2021), and often show micro zonation (Li and Bishop 2004; Nguyen et al. 2012). However, it remains elusive whether or not FLBA is a robust and conservative approach under all these environmentally relevant redox conditions. Combinations of FLBA with redox sensors, or with electrochemical methods are promising tools that allow for investigating reactions of microenvironments under changing redox conditions, or the role of EPS in controlling such redox microenvironments and, as a result, reaction mechanisms within a biofilm.

In this study, we investigate the suitability of two commonly used lectin-fluorophore conjugates for biofilm studies under varying, controlled redox conditions by using a combination of in situ CLSM and electrochemistry.

We hypothesize that FLBA can be applied to redox-active environmental biofilms to study the composition and distribution of EPS. Potential applications include the study of EPS functions for element cycling or the fate of toxic compounds.

## Materials and methods

### Sample preparation

Environmental, redox-active biofilm samples were collected using sterile 50 ml sample tubes. The biofilms sampled were from iron rich creeks located at Mount Rudolfstein and Mount Nusshardt (Bavaria, Germany).

Wheat germ agglutinin Alexa Fluor^™^ 555 (WGA555) and Concanavalin A Alexa Fluor^™^ 488 (ConA488) conjugates (Thermo Fisher Scientific) were used respectively to label biofilm glycoconjugates.

Each biofilm sample was stained by adding 10 µl of Alexa Fluor^™^ conjugate stock solution (1 mg/ml) to 100 µl of biofilm. Biofilms were incubated in the dark for 20 min before analysis, allowing for the quantitative binding of the fluorescence probes.

Two types of control samples were prepared. The respective fluorescence probe stock solution was either diluted in sterile filtered biofilm water or in sterile filtered biofilm water buffered with 0.1 M 2-(N-Morpholino)ethanesulfonic acid hydrate (MES, ≥ 99.5%, Sigma Aldrich) to a final concentration of 0.025 mg/ml.

Multi label biofilm staining for the exemplary biofilm study was done according to (Hao et al. 2016) using (1) ConA488, SYTO 62 (Thermo Fisher Scientific), and a Fe^2+^-Sensor (Kumar et al. 2011), and (2) ConA488, WGA555, and SYTO 40 (Thermo Fisher Scientific).

### Cell for electrochemical CLSM analysis

For the electrochemical CLSM measurements, a custom-made electrochemical measurement cell was used. A Frame was milled from Poly(methyl 2-methylpropenoate) with trenches for attaching electrodes. Gold wires were used as the working and counter electrodes (100 µm diameter, 99.998%, Alfa Aesar), which were secured in the trenches with glue (UltraGel, Pattex). The frame was sealed onto a microscope slide using silicone sealant (Dow Corning 3140). A pseudo reference electrode (Ag/AgCl) was prepared by anodic silver chloride coating silver wires. A protocol adapted from (Smith and Stevenson 2007) was used for this process. First, silver wires (250 µm diameter, 99.9985%, Alfa Aesar) were cleaned with ethanol. To get rid of the oxide layer the wires were dipped in 0.1 M HNO_3_ for 10 s. For anodic coating, the silver wires were immersed in 0.5 M HCl for 30 min with a voltage of 3.0 V applied between anode and cathode. The coated silver wires were washed with ultra-pure water (resistivity > 18.2 MΩ cm). For each experiment, a fresh electrode was prepared.

Before each use, the cell was cleaned electrochemically with 0.05 M H_2_SO_4_. After each use, the cell was filled with 30% hydrogen peroxide for 10 min to clean off organic residues and rinsed thoroughly with ultra-pure water.

### Electrochemical CLSM

The cell was filled with sterile-filtered biofilm supernatant water (0.2 µm PES membrane filter, VWR). Stained biofilms were placed close to the working electrode.

Electrochemical CLSM measurements were carried out using an upright Leica TCS SPE equipped with a 20x/NA 0.5 water immersion dipping lens (HCX APO L U-V-I UV, Leica Microsystems, Wetzlar, Germany). Measurements were taken at regions (area of 75.625 µm^2^) close to the working electrode. The pinhole was set to 2 AU to increase the depth of field in order to counteract the influence of potential z-movement of the sample within the time of analysis.

A time series experiment was setup where the applied potential was changed in 15 s intervals and an image was recorded at the end of each 15 s interval. Each potential step was repeated 3 times during the time series experiment. Each cycle was divided in two parts. First, the reduction potential was gradually lowered in an environmentally relevant range from + 0.2 to − 0.5 V. In order to repeat this cycle, an over potential was applied in three increments (from + 0.7 to + 1.1 V) to ensure re-oxidation of the redox-active compounds in the sample.

As a control, the fluorescence intensity of the biofilms was recorded in the same way at open circuit potential (OCP).

### Data analysis

The time series image stacks were analyzed using Fiji (Schindelin et al. 2012). Fluorescence intensity profiles over time were extracted by measuring the mean intensity at each time point. For the biofilm datasets, regions of interest (ROIs) were chosen to select in focus areas of the biofilm, as well as to account for eventual lateral movement of the sample during the measurement. ROIs were generated by applying an automated threshold (IsoData) on the maximum intensity projection of the image stack, smoothed with a median filter (r = 5 pixels).

Measurements were affected by bleaching, which was corrected for by fitting and dividing by an exponential decay baseline for each profile.

A one-way ANOVA was conducted to compare the effect of applied potential on fluorescence intensity.

## Results

The fluorescence intensity response of lectin-fluorophore conjugates to the applied potentials was studied in 3 different treatment conditions: (1) Biofilms, (2) filtered biofilm water, and (3) filtered biofilm water, buffered with 0.1 M MES.

**Fig. 1:**
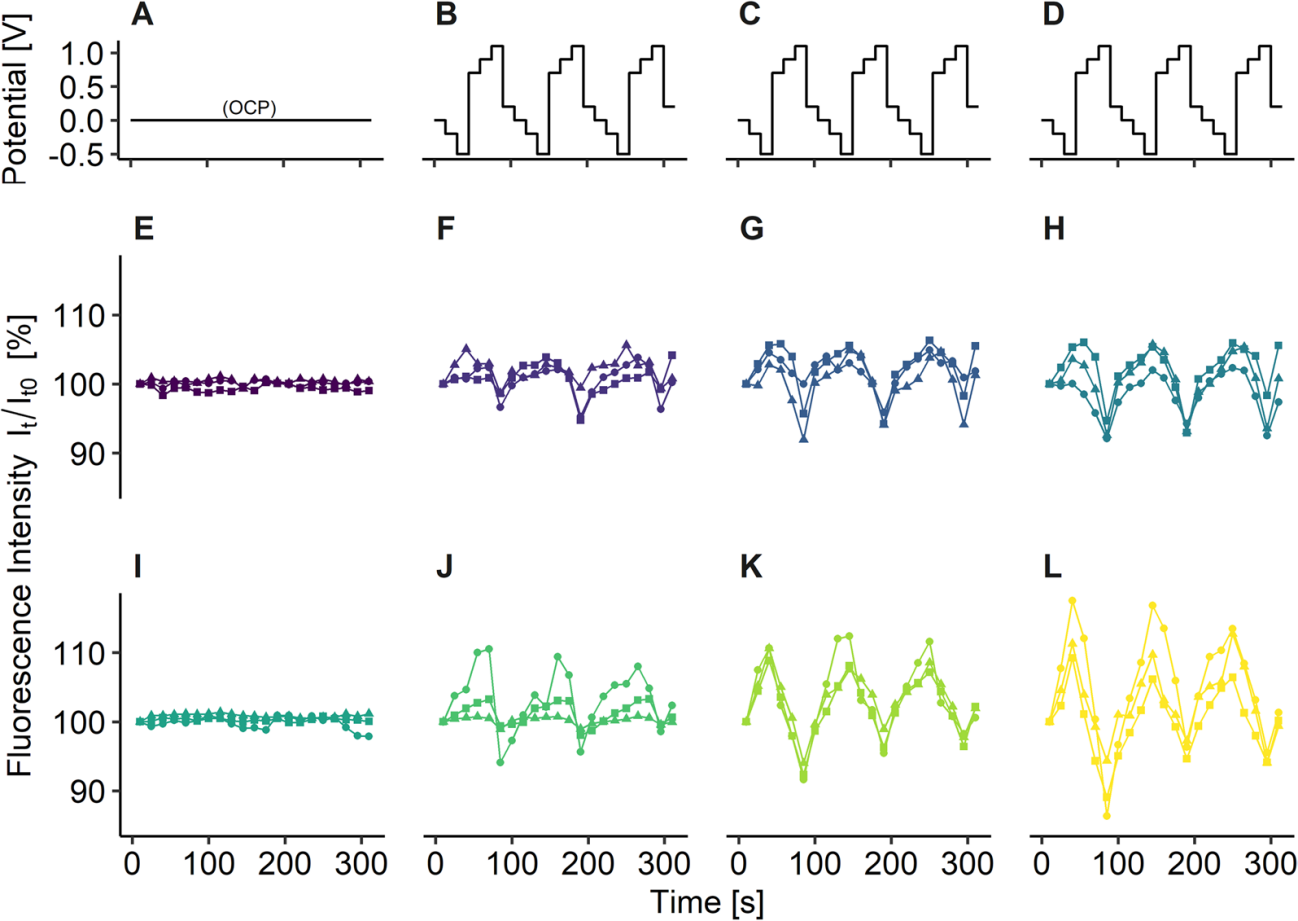
Electrochemical CLSM characterization of ConA488 and WGA555. Applied potential over time (**A**–**D**). Corresponding mean fluorescence intensity for ConA488 (**E**–**H**) and WGA555 (**I**–**L**). Columns from left to right show labeled biofilm without potential applied (**A**, **E**,** I**), labeled biofilm under potential control (**B**, **F**, **J**), fluorescence probe in filtered biofilm water (**C**, **G**, **K**), fluorescence probe in filtered biofilm water, buffered with 0.1 M MES (**D**, **H**, **L**). Potential/V vs. Ag/AgCl wire

The results are summarized in Fig. 1. In general, the fluorescence intensity of the lectin-fluorophore conjugates ConA488 and WGA555 changed with applied potentials for all treatments. These changes in intensity as a function of potential were small on an absolute scale, but statistically significant (Table 1) with p < 0.001 for all treatments. At OCP the fluorescence was stable over time (Fig. 1 A, E, I).

**Table 1:** Effect of potential on fluorescence intensity of ConA488 and WGA555. Summary of ANOVA results for each experimental setup

	Effect	DFn	DFd	F	p
ConA488					
(1)	Potential	6	56	13.0	3.71e−09
(2)				18.8	7.36e−12
(3)				22.2	3.63e−13
WGA555					
(1)	Potential	6	56	7.5	6.04e−06
(2)				22.8	2.03e−13
(3)				43.1	3.00e−19

Stained biofilm (1), fluorescence probe in filtered biofilm water (2), and fluorescence probe in filtered biofilm water, buffered with 0.1 M MES (3). Degrees of freedom in the numerator (DFn) and denominator (DFd), F-statistic (F) and P-Value (p)

The relative changes in fluorescence are summarized in Table 2. During the reductive part of the potential cycle, the applied potential was lowered incrementally from + 0.2 to − 0.5 V. During this reducing sweep, the fluorescence intensity steadily increased by a few percent with decreasing potential. For the following oxidative part of the cycle (+ 0.7 to + 1.1 V), the intensity decreased with each increase in the potential. The magnitude of intensity changes was higher in (2) and (3) as compared to (1) for both, ConA488 and WGA555.

**Table 2:** Mean fluorescence intensity change vs. intensity I_t0_ (%) at different potentials (V) of dyes ConA488 and WGA555 under different conditions

	ConA488			WGA555		
Potential	(1)	(2)	(3)	(1)	(2)	(3)
Reduction sweep						
0.2	0.8	1.5	0	− 0.1	0.9	0.0
0	0.8	1.5	0.7	0.6	2.8	2.2
− 0.2	1.6	2.4	1.8	2.0	6.5	5.7
− 0.5	2.7	4.6	3.9	2.2	9.5	11.5
Oxidizing sweep						
0.7	2.3	3.7	3.2	4.3	4.1	6.0
0.9	2.0	1.2	0.0	3.7	0.8	0.3
1.1	− 2.4	− 3.9	− 6.2	− 1.9	− 4.3	− 6.5

Stained biofilm (1), fluorescence probe in filtered biofilm water (2), and fluorescence probe in filtered biofilm water, buffered with 0.1 M MES (3). Potentials are listed in sequence of appearance during cycling

When switching from the reduction to the oxidation sweep a hysteresis effect was observed for (1). A significant drop in fluorescence intensity showed only after a lag phase of 30 s at a potential of + 1.1 V.

**Fig. 2:**
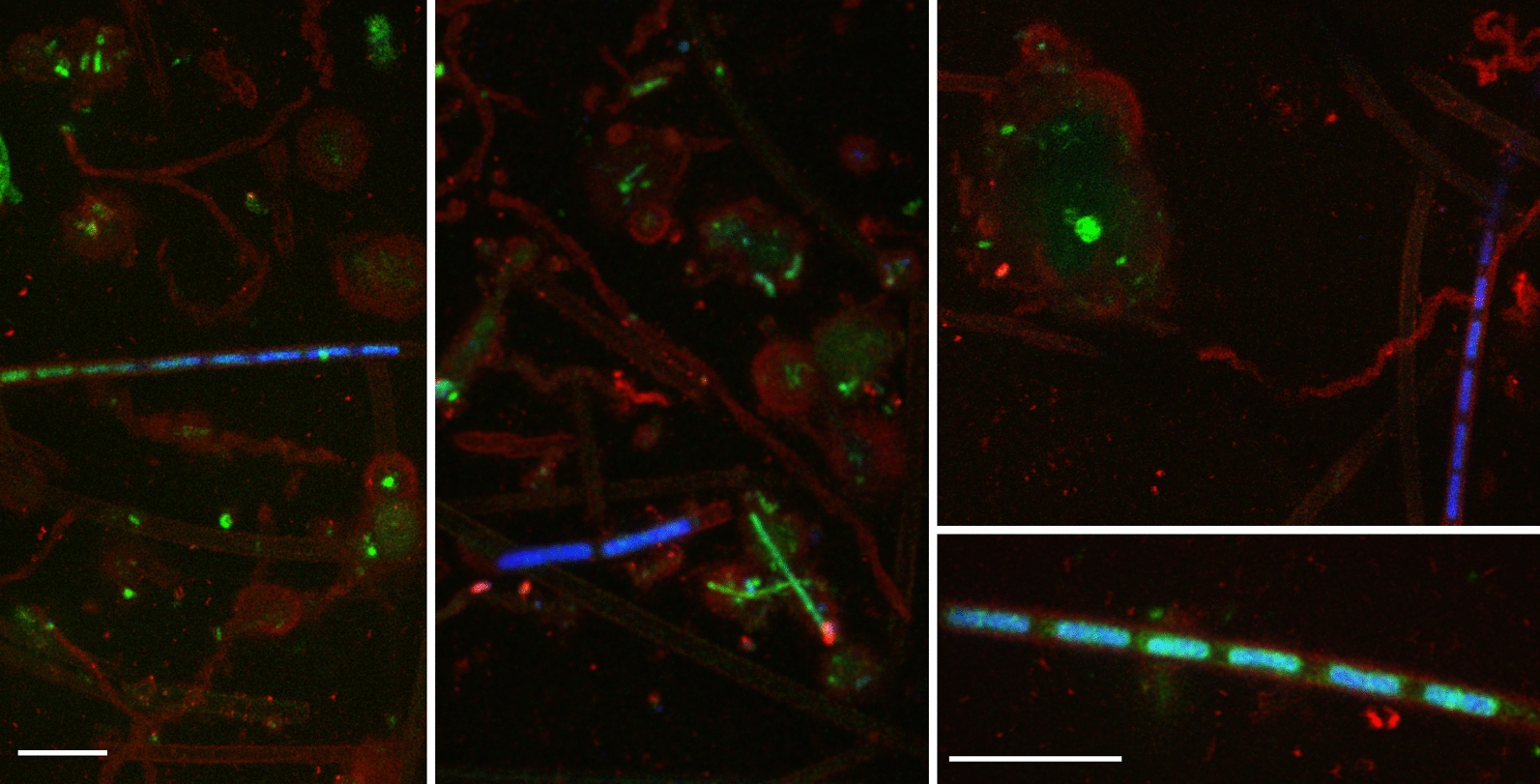
Exemplary Staining of a redox-active environmental Biofilm containing iron oxidizing bacteria. Red: EPS (ConA488), green: Fe^2+^ (Fe^2+^-Sensor), blue: DNA (Syto 62). Scalebar 10 µm

Figure 2 shows several examples of a redox-active environmental biofilm, sampled from a Fe-rich creek, with a pH of 6.6 and O_2_ concentration of 1.4 mg/L (T = 12.5 ℃). The biofilm was stained by a combination of three dyes visualizing the DNA of microbial cells (blue), the EPS of the biofilm via FLBA (red) and the distribution of dissolved Fe^2+^ (green). The figure visualizes exemplarily the existence of microenvironments in the biofilm that are enclosed by the EPS and enriched in dissolved Fe^2+^ ions. Additional 3D representations of such biofilms stained with different dye combinations can be found in the Additional file 1 (Fig. S1).

## Discussion

We hypothesize that FLBA is both robust and conservative under environmentally relevant redox conditions. By in-situ electrochemical confocal laser scanning microscopy, we clearly show that under environmentally relevant redox conditions, the fluorescence intensity of the two tested lectin Alexa Fluor conjugates vary in the low percent range. These differences were significant on a p < 0.0001 level, reproducible and reversible after several electrochemical reoxidation cycles.

Our results on lectin Alexa Fluor conjugates are in contrast to previous studies wherein other organic fluorophores showed much larger fluorescence intensity changes as a function of redox potential (Lei et al. 2009; Salverda et al. 2010). The Alexa Fluor dyes used in our study appear to be far less sensitive to changes in potential. Furthermore, the large decrease in fluorescence intensity was only expected for very extreme, oxidizing redox conditions (i.e. + 1.1 V), which are typically not found in environmental biofilms.

For FLBA in combination with CLSM, the observed fluorescence intensity at certain biofilm locations correspond to the amount of bound lectin at the respective location. A higher abundance of available binding sites within the biofilm EPS results in the binding of more lectin-fluorophore conjugates and, as a result, higher observed fluorescence intensity at this site and vice versa.

Staining of the biofilm with the lectin conjugates ConA488 and WGA555 indicates the presence of α-D-mannopyranosyl and α-d-glucopyranosyl (Goldstein et al. 1974), and N-acetylglucosamine and N-acetylgalactosamine (Wright 1984), respectively. This agrees with a study on similar biofilms (Takeda et al. 2005) showing that the EPS of a comparable metal-rich aquatic biofilm contained glycine, cysteine, galactosamine, glucosamine, and uronic acids. These sugar residues contain polar functional groups such as hydroxyls and amines, which are essential for the binding specificity of the lectins (Lis and Sharon 1986). If these polar groups of the EPS were chemically altered by changing redox potentials, this would change the amount of lectin conjugate bound in the biofilm. As a result, the observed fluorescence intensity of the bound lectin-fluorophores would be expected to strongly decrease. However, in our study the signal intensity remains unaffected by redox potentials within the range of a few percent.

Thus, the small fluorescence intensity changes [i.e. 4.6% at max in an environmentally relevant potential range (Zobell 1975) between − 0.5 and + 0.7 V] observed in our study could in principle be caused either by decreased/increased binding of lectin-fluorophore conjugate to the respective mono- or oligosaccharide moieties, or by an oxidation/reduction of the fluorophore itself.

The comparison of our results on the effect on fluorescence intensity of the lectin-fluorophore conjugates in aqueous solution (with and without pH buffer), and the same conjugates bound to the EPS structures clearly indicates that the intensity changes are caused by the fluorophore itself, and not the binding of the lectin to the target sites. The fluorescence intensity changes of the EPS-bound lectin Alexa Fluor conjugates were small compared to the dye-conjugates in the buffered or non-buffered aqueous solutions (compare Fig. 1 and Table 2).

Therefore, FLBA with the two tested lectin Alexa Fluor conjugates was demonstrated to be a robust and conservative approach for studying the distribution and composition of EPS in environmental biofilms under various and under varying redox conditions.

The relevance of these findings are manifold. The stained biofilm shown in Fig. 2 visualizes exemplarily the potential of FLBA to gain mechanistic insights into the processes within and functions of environmental biofilms. FLBA was used to identify microbial processes resulting in redox microzonation. Although it is not possible to quantitatively measure the redox potential at the µm scale, there is clear indirect evidence for heterogeneity and redox-microzonation at this scale. The enrichment of dissolved or weakly sorbed Fe^2+^ ions within the globular EPS structures, particularly around the microbial cells in the center of these structures indicates the presence of such reduced microzones, as it was expected that Fe^2+^ would be readily oxidized at the circumneutral pH of 6.6 within the biofilm under oxic or suboxic conditions. Simultaneously with the increase of the fluorescence signal of the Fe^2+^ sensitive probe towards the interior of the round EPS structures, we observed a strong decrease in the ConA488 signal, which was attribute to a change in composition of the EPS structures surrounding the microorganisms and not to the redox-potential on ConA488 itself. Through this project, we were able to identify transitions in the organochemical composition of EPS compounds under varying redox potentials within the microzonation of a biofilm. We clearly demonstrated that the differences in the fluorescence intensity of ConA488 between the inside and the outside of the Fe^2+^-rich microzones presented in Fig. 2, cannot be caused by effects of associated redox-zonation on ConA488, but must be caused by compositional variations within the EPS.

Our results are also relevant for various other studies of biofilm functions as for instance microbial “communication” in biofilms. Bacteria within a biofilm were shown to communicate by transmitting electrical impulses through potassium ion channels within the EPS matrix (Czerwińska-Główka and Krukiewicz 2020).

Both, communication and electroactivity for metabolism or nutrient acquisition requires electron transfer to and from the microorganisms. This can be achieved by small organic redox-active molecules, such as flavins and cytochromes on the cell-surface (Flemming 2011; Obst et al. 2018; Xiao et al. 2017), that act as electron shuttles (Hernandez and Newman 2001), conductive microbial structures such as pili or even electrically conductive microbial lifeforms such as cable bacteria. The latter, for example, are capable of electron transport over long distances and directly couple processes across redox gradients spanning from the oxic to sulfidic zone (Bjerg et al. 2018; Müller et al. 2016; Nielsen et al. 2010).

The approach of this study successfully acquired real-time imaging of fluorescent-labelled EPS under varying redox conditions. It can be used for non-destructive in situ biofilm studies on various properties of microbial EPS that are relevant for processes such as element cycling or for the fate of nutrients or toxic compounds (e.g., metal sorption as a mechanism to immobilize pollutants). The spatial distribution of the sorption of heavy metals by EPS was demonstrated previously by metal-specific fluorescence probes (Hao et al. 2016). This process was directly affected by changing redox conditions and the spatial heterogeneity in the organochemical composition of the EPS can now be studied efficiently by FLBA.

Therefore, the results of our study are not only relevant for environmental biofilms but also for the fast emerging field of technical applications of biofilms, such as the use of Fe(II)-oxidizing biofilms as efficient filter materials for metal contaminated mine drainage (Hedrich and Johnson 2014; Janneck et al. 2010). Other potential fields of relevance include the growth of biofilms on electrodes for future use in technical and industrial applications such as microbial fuel cells, an emerging technology (Kumar et al. 2018) where FLBA could lead to a better understanding of electroactive biofilm formation and electrode interaction. However, for some applications, further aspects might have to be considered.

For example, Babauta et al. (Babauta et al. 2012) studied redox potential variations within a growing *G. sulfurreducens* biofilm on a larger spatial scale using microelectrodes. They elucidated that biofilms grown under electrode-respiring conditions show an increase in redox potential and decrease in pH with increasing distance from the electrode. In such biofilms, heterogeneities in EPS structure and composition are expected and may be related to local biofilm redox potentials. Beyond the local redox potential, the local pH in a biofilm may also affect either the lectin-EPS binding behavior or the fluorescence of the fluorophore itself. In our case, this is not critical as Alexa Fluor dyes are stable over a pH range of 4 to 9 (Panchuk-Voloshina et al. 1999) and the ligand binding optima of WGA (Privat et al. 1974) and ConA (Hassing and Goldstein 1970) are in the pH range of 4 to 10 and 3 to 8, respectively. However, pH should still generally be considered as an important parameter in the discussion and interpretation of lectin binding in microbial biofilms and the influence of local pH changes so far remains elusive.

For the lectin-fluorophores used in this study, we demonstrated and discussed their usability over a wide range of redox potentials and pHs. One should note however, that this would not be universally applicable to all lectin Alexa Fluor combinations. For example, redox dependent photo switching was recently shown for Alexa Fluor 647 (Fan et al. 2019). The present study can act as a guideline in evaluating the suitability of other lectin-fluorophore conjugates for FLBA studies under varying and in various redox environments.

The approach of electrochemical CLSM in combination with FLBA that was developed for this study allows for non-destructive in situ biofilm studies under controlled redox conditions. FLBA using lectin Alexa Fluor conjugates has been demonstrated to be a robust and conservative technique to study biofilm composition under varying redox-conditions. Future environmental and technical applications may include studies on metal sorption as a mechanism to immobilize pollutants.

## Supplementary Information

**Additional file 1: Fig. S1.** 3D representations of environmental biofilms imaged by CLSM (Hao et al. 2016). A-B showing distribution of EPS, cells, and Fe2+. Red: EPS (ConA488), green: Fe2+ [Fe2+-Sensor (Kumar et al. 2011)], blue: DNA (Syto 62). C-D EPS heterogeneity shown by use of the two lectin stains investigated in this study. Red: EPS (ConA488), green: EPS (WGA555), blue: DNA (Syto 40). Scalebar 20 μm.

## Data Availability

All data analyzed during this study were included in this article and microscopy raw data would be made available upon reasonable request.
