# Supplementary material for: Suitability of lectin binding studies for the characterization of redox-active microbial environmental biofilms
Source: AMB Express. 2022 Nov 5;12:140. doi: 10.1186/s13568-022-01479-7 (PMC9637051; doi:10.1186/s13568-022-01479-7)
Supplement: Supplementary file 1 — Additional file 1: Fig. S1. 3D representations of environmental biofilms imaged by CLSM (Hao et al. 2016). A-B showing distribution of EPS, cells, and Fe2+. Red: EPS (ConA488), green: Fe2+ [Fe2+-Sensor (Kumar et al. 2011)], blue: DNA (Syto 62). C-D EPS heterogeneity shown by use of the two lectin stains investigated in this study. Red: EPS (ConA488), green: EPS (WGA555), blue: DNA (Syto 40). Scalebar 20 μm. [file 13568_2022_1479_MOESM1_ESM.pdf]

Supporting Information

Suitability of lectin binding studies for the characterization of redox-active microbial environmental biofilms

Pablo Ingino[1,*], Kai Hao Tiew[2], Martin Obst[1]

[1] BayCEER, University of Bayreuth, D-95448 Bayreuth, Germany

[2] School of Science, Monash University Malaysia, Subang Jaya, Malaysia

[*]corresponding author

University of Bayreuth
BayCEER
Dr-Hans-Frisch-Str. 1-3
95448 Bayreuth
pablo.ingino@uni-bayreuth.de

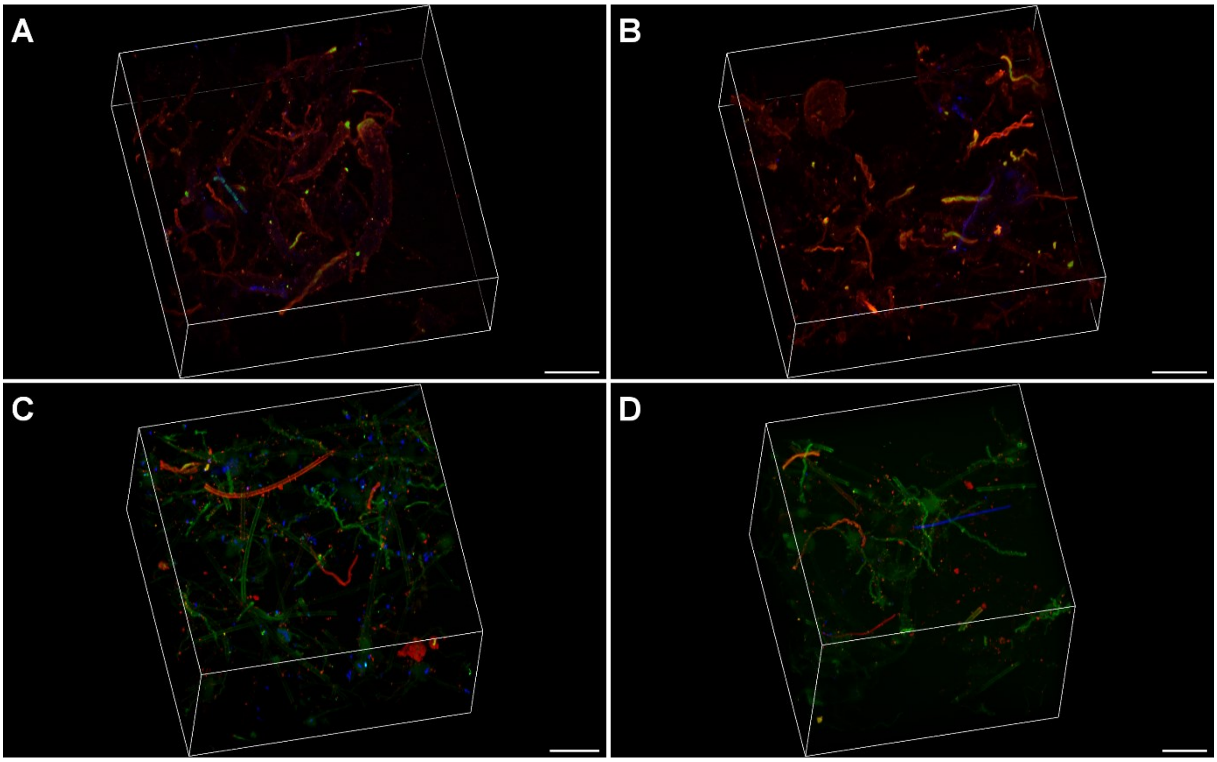

Figure S1: 3D representations of environmental biofilms imaged by CLSM (Hao et al. 2016). **A-B** showing distribution of EPS, cells, and Fe^{2+}. Red: EPS (ConA488), green: Fe^{2+} (Fe^{2+}-Sensor (Kumar et al. 2011)), blue: DNA (Syto 62). **C-D** EPS heterogeneity shown by use of the two lectin stains investigated in this study. Red: EPS (ConA488), green: EPS (WGA555), blue: DNA (Syto 40). Scalebar 20 µm.

References

Hao LK, Guo Y, Byrne JM, Zeitvogel F, Schmid G, Ingino P, Li JL, Neu TR, Swanner ED, Kappler A, Obst M (2016) Binding of heavy metal ions in aggregates of microbial cells, EPS and biogenic iron minerals measured in-situ using metal- and glycoconjugates-specific fluorophores. Geochim Cosmochim Acta 180:66-96 doi:10.1016/j.gca.2016.02.016

Kumar M, Kumar N, Bhalla V (2011) FRET-induced nanomolar detection of Fe2+ based on cinnamaldehyde-rhodamine derivative. Tetrahedron Lett 52(33):4333-4336 doi:doi.org/10.1016/j.tetlet.2011.06.044
